# GONAD: *G*enome-editing via *O*viductal *N*ucleic *A*cids *D*elivery system: a novel microinjection independent genome engineering method in mice

**DOI:** 10.1038/srep11406

**Published:** 2015-06-22

**Authors:** Gou Takahashi, Channabasavaiah B Gurumurthy, Kenta Wada, Hiromi Miura, Masahiro Sato, Masato Ohtsuka

**Affiliations:** 1 Department of Molecular Life Science, Division of Basic Medical Science and Molecular Medicine, Tokai University School of Medicine, 143 Shimokasuya, Isehara, Kanagawa 259-1193, Japan; 2 Mouse Genome Engineering Core Facility, Department of Genetics, Cell Biology and Anatomy, University of Nebraska Medical Center, Omaha, NE, 68198, USA; 3 Department of Bioproduction, Tokyo University of Agriculture, 196 Yasaka, Abashiri, Hokkaido, 099-2493, Japan; 4 Section of Gene Expression Regulation, Frontier Science Research Center, Kagoshima University, 8-35-1 Sakuragaoka, Kagoshima, Kagoshima 890-8544, Japan

## Abstract

Microinjection is considered the gold standard technique for delivery of nucleic acids (NAs; transgenes or genome editing tools such as CRISPR/Cas9 systems) into embryos, for creating genetically modified organisms. It requires sophisticated equipment as wel as well-trained and highly skilled personnel to perform the micro-injection technique. Here, we describe a novel and simple microinjection-independent technique, called Genome-editing via Oviductal Nucleic Acids Delivery (GONAD). Using GONAD, we show that NAs (e.g., eGFP mRNA or Cas9 mRNA/sgRNAs) can be effectively delivered to pre-implantation embryos within the intact mouse oviduct by a simple electroporation method, and result in the desired genetic modification in the embryos. Thus GONAD can bypass many complex steps in transgenic technology such as isolation of zygotes, microinjection of NAs into them, and their subsequent transfer to pseudo-pregnant animals. Furthermore, this method can potentially be used for genome editing in species other than mice.

Generally, the creation of genetically engineered animals involves modifying their genomes at early embryonic stages. This complex process consists of three major and absolutely critical steps: i) isolation of zygotes from super-ovulated females, ii) delivery of nucleic acids (NAs) into the isolated zygotes and iii) subsequent embryo transfer into pseudo-pregnant animals to produce viable offspring. For optimal success, these steps require sophisticated equipment and a series of timely, and well-planned procedures, performed by experienced and skilled personnel. While isolation and transfer of embryos are surgical procedures, the delivery of NAs into embryos is performed predominantly through microinjection. There are other less commonly used methods to deliver NAs, such as electroporation-mediated gene transfer, viral transduction using adeno-, retro- and lentiviral vectors, and liposomal transfection (reviewed in Smith, 2004^1^).

In order to simplify the genetic engineering procedures, we and others have been developing alternate methods that would help circumvent some or all of the three major steps listed above. Two years ago, we demonstrated that naked plasmid DNA can be instilled into the mouse oviductal lumen at day 1.5 of gestation, which was successfully electroporated into 2-cell stage embryos present within the oviduct^2^. The DNA solution was instilled using a glass micropipette and tweezer-type electrodes were used for electroporation. Since the process was done on the exposed oviduct *in situ* in the anesthetized mouse and the embryos developed in the same mouse, we bypassed the first step of embryo isolation and did not need the third step of embryo transfer. Thus, by using electroporation instead of microinjection, we were able to circumvent the three critical steps outlined above. Upon electroporation, about 30–60% of embryos took up the plasmid DNA^2^. Even though we demonstrated that plasmid DNA can be delivered to embryos within the oviduct *in situ* by electroporation, the offspring we created were not genetically modified in the true sense because the plasmid DNA did not get integrated into the genome^2^.

The CRISPR/Cas9 system is a recently developed novel genome-editing tool that is used for targeted genome modification^3^,^4^. Recently, another group (Kaneko *et al.*, 2014) used electroporation (instead of microinjection) to deliver CRISPR/Cas9 reagents into isolated rat embryos to generate mutant rats^5^. However, their procedure only bypassed the microinjection step and not the embryo-isolation and embryo-transfer steps.

In this work, we have developed a new method called Genome-editing via Oviductal Nucleic Acids Delivery (GONAD) that can bypass all three steps of embryo isolation, microinjection and embryo transfer. Using GONAD, we demonstrate that CRISPR/Cas9-mediated targeted genome editing can be performed without the need for *ex vivo* handling of embryos. This is the first report of a method that can circumvent all three major steps of animal transgenesis.

## Results

### Electroporation of mRNA into 2-cell embryos within dissected oviducts

**Figure 1:**
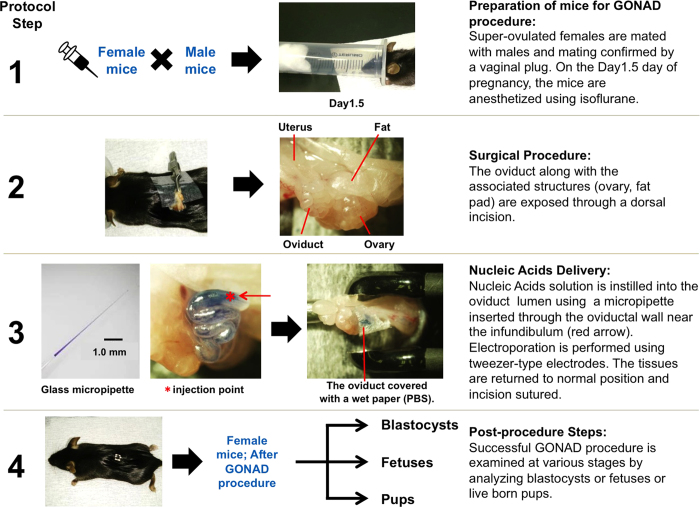
Overview of the Genome-editing via Oviductal Nucleic Acids Delivery (GONAD) system.

We examined whether the eGFP mRNA introduced into oviductal lumen can be transferred to zona-intact 2-cell embryos, and result in green fluorescence. To test the possibility, we first dissected oviducts from super-ovulated pregnant females (corresponding to 2-cell stage), and instilled 2 μL of solution containing eGFP mRNA (500 ng/μL) and 0.05% trypan blue (to aid in visualization of the injected solution) into the lumen of the dissected oviducts, as depicted in step3 of Fig. 1. The oviducts were then placed in a cuvette with a 1-mm gap, and electroporated at 50 V. The embryos were then flushed from the treated oviducts and cultured for 2 to 3 days in KSOM medium. Inspection of developing embryos under a fluorescence stereomicroscope revealed that 25% (4/16) of normally developed embryos exhibited fluorescence (Supplementary Fig. S1 and Table S1). This demonstrates that intra-oviductal administration of mRNA and subsequent electroporation enables successful delivery of mRNA to the zona-intact pre-implantation embryos floating in the oviductal lumen. Further, the mRNA thus introduced inside the embryos is translated to functional protein.

### Electroporation of mRNA into 2-cell embryos within oviducts *in situ*

**Figure 2:**
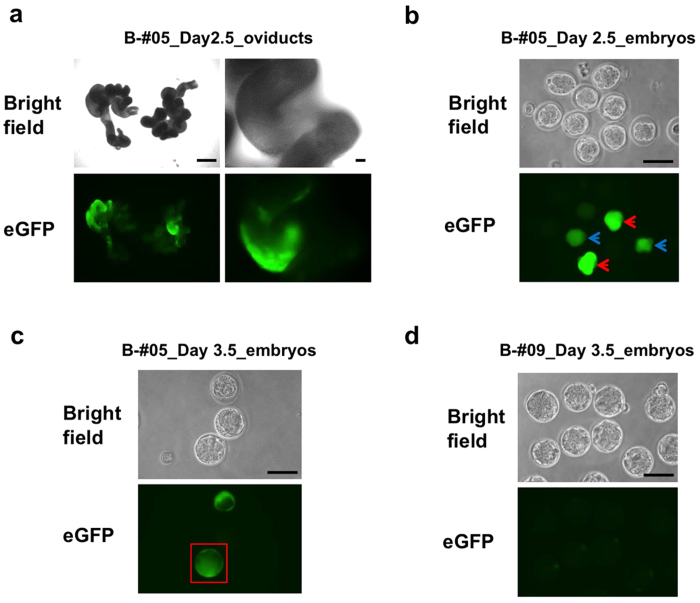
eGFP expression in the oviducts and embryos following the GONAD procedure. (**a**) eGFP fluorescence in the oviducts dissected and analyzed one day after the procedure (from mice B-#05, see Supplementary Table S2). (**b**) eGFP fluorescence in 8- to 16-cell stage embryos recovered from dissected oviducts shown in (a). The microscopic field shows the representative zygotes with bright (red arrows) and moderate (blue arrows) eGFP fluorescence. (**c**) eGFP fluorescence in blastocysts derived from embryos in (b) after one day-culturing, showing a normal looking embryo with uniform fluorescence (red box). (**d**) eGFP fluorescence in morula to blastocyst stage control embryos recovered from a super-ovulated female not subjected to GONAD procedure (B-#09). Scale bars = 1 mm (left side panel in a), 100 μm (right side panels in a, and b to d).

Next, we examined whether a similar approach can be used directly on intact oviducts *in situ* (without dissecting them out from the mouse) for delivering mRNA to 2-cell staged embryos. We first instilled approximately 2 μL of solution containing eGFP mRNA (500 ng/μL) and 0.05% trypan blue into the oviductal lumen of super-ovulated pregnant females (corresponding to 2-cell stage) and then electroporated them as described in Methods. The experiment was performed on six mice (total of 12 oviducts). The oviducts were then isolated from the mice next day and examined for fluorescence (as described in Sato *et al.*, 2012^2^). As shown in Fig. 2a and Supplementary Table S2, all oviducts fluoresced although the intensity of fluorescence and the areas covered by the fluorescence signal differed among oviducts. The embryos were flushed out and were examined for the presence of fluorescence. A total of 52 embryos were recovered, of which 23 were normal while the remaining 29 showed abnormal morphology. Of the total 23 normal embryos, 6 (26.1%) were fluorescent with different degrees of fluorescence. Representative examples of embryos with different transfection efficiencies are shown in Fig. 2b. Some embryos exhibited bright fluorescence (red arrows in Fig. 2b), while some showed moderate fluorescence (blue arrows in Fig. 2b). In contrast, other embryos were non-fluorescent (or the intensity was lower than the detection limit). Notably, the inner cell mass (ICM) of some blastocysts were uniformly fluorescent (shown in a red box in Fig. 2c). As expected, the embryos from control samples (no DNA instillation and no electroporation) were negative for eGFP-derived fluorescence (Fig. 2d). These results demonstrate that mRNAs can be delivered directly to 2-cell staged embryos within the oviduct *in situ*.

### Electroporation of CRISPR/Cas9 components into 2-cell embryos within dissected oviducts

Next, we tested if CRISPR/Cas9 tools can be introduced into embryos by electroporation to achieve genome editing. We injected CRISPR/Cas9 RNAs into the lumen of dissected oviducts containing 2-cell embryos and subjected them to electroporation. The eGFP mRNA was included in some experiments to serve as an indicator of successful electroporation. We used two endogenous (*Hprt* and *eEF2*) and one exogenous (eGFP) loci and a total of four sgRNA targets; one target site in an upstream region of *hypoxanthine guanine phosphoribosyl transferase* (*Hprt*) (Hprt_Cr1), two target sites in the introns of *eukaryotic translation elongation factor* 2 (*eEF2*) gene (eEF2_Cr1 and Cr2) and one site targeting eGFP gene (Supplementary Table S3). While wild-type mice were used as oviduct donors for endogenous targets, eGFP Tg mice containing a single-copy eGFP expression cassette in the *Rosa26* locus (eGFP is ubiquitously expressed in all fetal tissues)^6^ was the oviduct donor for the eGFP locus. Embryos were flushed from the treated oviducts and then cultured up to blastocysts stage. Genomic DNA was isolated from the blastocysts and examined for the presence of mutations in the target sites using either T7 endonuclease 1 (T7E1) or Surveyor assay and followed by sequencing. The results are presented in Supplementary Figures S2, S3, S4 and Supplementary Table S3. The *indel*s (*in*sertion or *del*etion) mutations were detected in at least 1 embryo from each of the target genes (2 at *Hprt* locus, 2 at *eEF2*_Cr1 locus and 1 at eGFP locus). Of note, the experiments where eGFP mRNA was included as an indicator of electroporation, the *indel*s were detected only in the eGFP positive embryos suggesting that eGFP fluorescence can serve as an indicator of successful electrophoretic delivery of reagents (Supplementary Fig. S2).

Interestingly, when eGFP sequence was targeted, the precise targeting of the eGFP locus resulted in embryo with diminished eGFP expression as expected (embryo from C-#13 mouse in Supplementary Fig. S4 and Table S3). These results demonstrate that intra-oviductal injection of CRISPR/Cas9-related RNAs and subsequent *in vitro* electroporation can induce targeted mutations in the pre-implantation embryos although with low efficiency. Insufficient concentration of Cas9 mRNA and/or sgRNA could have resulted in the lower efficiency of targeting, which was addressed in the experiment below. Nevertheless, the *indel*s were detected in three of the four sgRNAs that we tried.

### Electroporation of CRISPR/Cas9 components into 2-cell embryos within oviducts *in situ*

We examined whether CRISPR/Cas9 - mediated genome editing among the 2-cell staged embryos can be done by direct electroporation of intact oviducts *in situ* (without separating them out from the mouse). As we observed lower efficiency of *indel* mutations in the previous experiment, we increased the concentrations of RNA (approximately twice) in this experiment (see Supplementary Table S3 and Table 1). One day after the procedure, the embryos were isolated and cultured for up to blastocyst stage and the genomic DNAs were isolated for mutation analysis at the target sites using T7E1-based assay and sequencing. Of a total of 6 mice were injected with Cas9 mRNA, Hprt_Cr1 sgRNA and eGFP mRNA, embryos isolated from five mice showed fluorescence (Table 1). Among the 49 embryos that developed normally to morula or blastocyst stage, 6 exhibited eGFP fluorescence (red arrows in Supplementary Fig. S5a). The T7E1-based assay and subsequent sequencing revealed that 5 of these embryos exhibited *indel* mutations at the target locus (Supplementary Fig. S5b,c). As observed in the previous experiments, the *indel* mutations were detected only among the embryos showing eGFP fluorescence.

**Figure 3:**
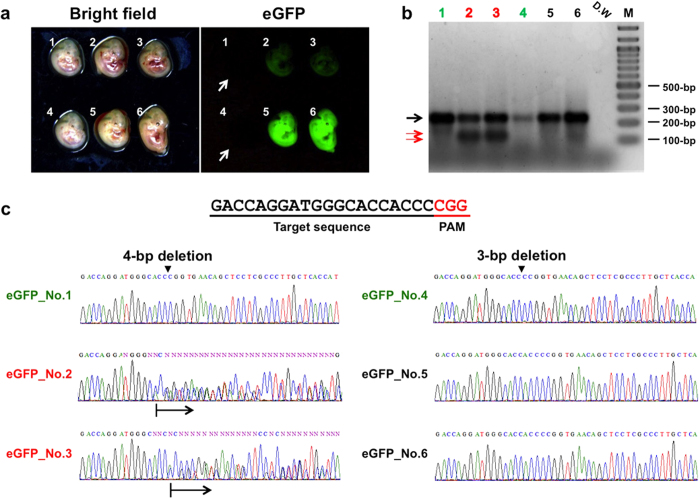
CRISPR/Cas9 - mediated genome editing using the GONAD system (day 13.5 fetuses). (**a**) The fetuses were isolated from one mouse at day 13.5 and analyzed for eGFP fluorescence using a fluorescence stereomicroscope. The fetuses 1 and 4 that lost eGFP expression completely (white arrows), the fetuses 2 and 3 exhibited greatly reduced eGFP expression and fetuses 5 and 6 had no loss of eGFP expression. (**b**) Agarose gel electrophoresis of T7E1-treated PCR products derived from fetuses shown in (a). The red arrows indicate the cleavage products generated in T7E1 assay and the wild-type sized band is indicated by a black arrow. D.W: distilled water used as a negative control. M: 100-bp DNA ladder marker. (**c**) Direct sequencing of PCR products amplified from the eGFP target region of all six fetuses. The black arrows below the electropherogram (in samples 2 and 3) show overlapping peaks indicative of *indel* mutations (see text for details). Note that there are three kinds of sequences: clear deletions (samples 1 and 4), mixed *indels* (samples 2 and 3) and no mutations (samples 5 and 6).

From all our experiments so far, when electroporated embryos were examined at blastocyst or morula stage, approximately 50% showed abnormal development (Table 1 and Supplementary Table S2). We believe that the reason for such abnormal development could be mainly due to the electroporation shock/damage because most of the control embryos (that did not receive electroporation treatment) developed normally. Such loss of embryos due to electroporation damage is an important factor to keep in mind when the pregnancy is allowed to continue. To assess embryo survival and implantation at mid-gestation, pregnancy was continued till day 13.5 after electroporation with Cas9 mRNA and eGFP sgRNA into eGFP Tg mouse line^6^. We could isolate a total of 14 fetuses (8 from left horn and 6 from right horn) in one experiment, which suggests that *in vivo* electroporated embryos can implant and develop further. All of the 8 fetuses that were recovered from the left uterus showed bright eGFP fluorescence indicating that either the electroporation or gene modification was unsuccessful. On the other hand, some fetuses isolated from the right horn showed obvious loss of eGFP fluorescence. Of the total six fetuses recovered, fetuses 1 and 4 exhibited complete loss of fluorescence (white arrows in Fig. 3a), fetuses 2 and 3 had reduced eGFP expression, while fetuses 5 and 6 exhibited normal eGFP fluorescence (Fig. 3a). Interestingly, the T7E1 assay showed results that were in agreement with the loss of eGFP expression for all fetuses except for 1 and 4 (see below). The fetuses 2 and 3 had reduced eGFP loss and showed expected cleavage bands of 108 and 122 bp in the T7E1 assay suggesting the possibility of mosaic *indel* mutations (Fig. 3b). The direct sequencing reactions exhibited mixed chromatogram^4^ in these fetuses suggesting that the mutations indeed were mosaic (Fig. 3c). The sequencing of cloned fragment revealed that one each of mutant (6-bp deletion [10/18; number of clones with mutation/number of clones sequenced] and 3-bp [3/10] deletion for fetuses 2 and 3, respectively) and wild-type alleles in both these samples (Supplementary Fig. S6). The fetuses 5 and 6 did not show loss of eGFP expression and they were negative in T7E1 assay which is also as expected. Sequencing of these samples confirmed that the target sites were unchanged [contained wild-type eGFP sequence (Fig. 3c)]. On the other hand, the fetuses 1 and 4 did not show expected positive results in T7E1 assay even though they had complete loss of eGFP expression (Fig. 3b). Sequencing of the PCR-amplified products from target sites revealed micro-deletions of 4- and 3-bp in fetuses 1 and 4 respectively (Fig. 3c and Supplementary Fig. S6).

**Figure 4:**
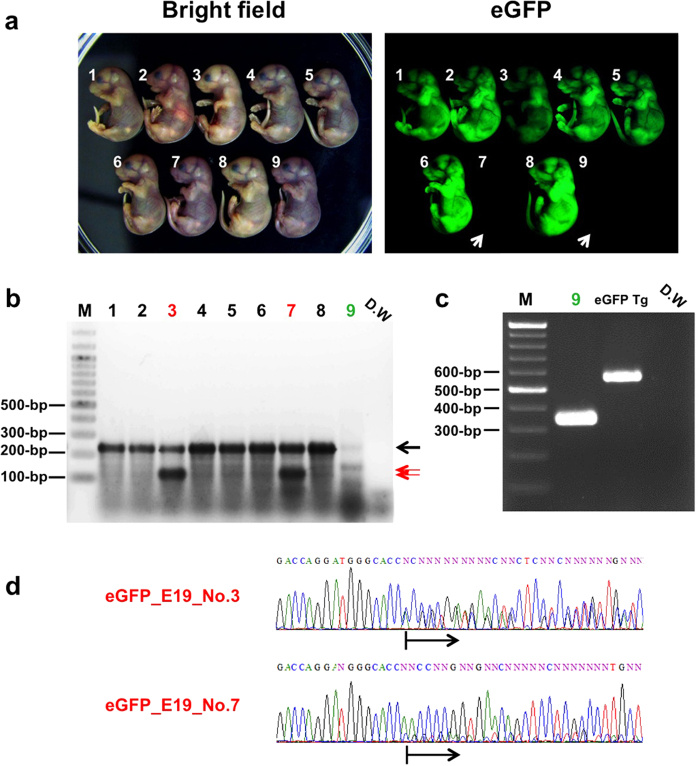
CRISPR/Cas9 - mediated genome editing using the GONAD system (day 19.5 fetuses). (**a**) The fetuses were isolated from one mouse at day 19.5 and analyzed for eGFP fluorescence using a fluorescence stereomicroscope. The fetuses 7 and 9 that lost eGFP expression completely (white arrows), the fetus 3 exhibited reduced eGFP expression and other fetuses had no obvious loss of eGFP expression. (**b**) Agarose gel electrophoresis of T7E1-treated PCR products derived from fetuses shown in (a). The red arrows indicate the cleavage products generated in T7E1 assay and the wild-type sized band is indicated by a black arrow. D.W: distilled water used as a negative control. M: 100-bp DNA ladder marker. (**c**) Agarose gel electrophoresis of PCR product amplified with primer set (M026: GGTGGTGCAGATGAACTTCAG and #125: CGGGATCCATTGCCTTTTATGGTAATCG) using genomic DNAs isolated from fetus 9 and eGFP transgenic mouse as template. (**d**) Direct sequencing of PCR products amplified from the eGFP target region of fetuses 3 and 7. The black arrows below the electropherogram show overlapping peaks indicative of *indel* mutations (see text for details).

We conducted another GONAD experiment to verify if the fetuses survive much longer period and nearly to the full term (day 19.5). The procedure was performed on four females of which one female retained pregnancy and we harvested the embryos at day 19.5 from this animal. We could isolate a total of 11 pups (2 from left horn and 9 from right horn). Both pups recovered from the left uterus showed bright eGFP fluorescence indicating that either the electroporation or gene modification was unsuccessful. On the other hand, two pups isolated from the right horn showed obvious loss of eGFP fluorescence (#7 and #9; white arrows in Fig. 4a) and one pup (#3) had reduced eGFP fluorescence (Fig. 4a). The T7E1 assay revealed that pups 3 and 7 contained possible *indels*, whereas the target site of pup 9 could not be amplified using this primer set (Fig. 4b). However, PCR of this sample using another set of primers that bind farther away (than the first set) from the Cas9 cut site successfully amplified a fragment. This result indicated that pup 9 contained approximately 200-bp deletion (Fig. 4c). We performed direct sequencing reaction of PCR products from the pups 3 and 7, which exhibited mixed chromatogram suggesting that these mutations were mosaic (Fig. 4d). Careful analysis of chromatograms revealed that the pup 3 had a 3-bp deletion in one allele and the other allele was wild-type whereas the pup 7 had 1-bp insertion and 4-bp deletions and lacked wild-type allele (Supplementary Fig. S6).

In summary, these results demonstrate that NAs can be successfully delivered to pre-implantation embryos through *in situ* electroporation without taking them out from the female reproductive tract. Furthermore, we demonstrate that targeted gene modification can be achieved when CRISPR/Cas9 tools are thus delivered. We have named this novel method “Genome-editing via Oviductal Nucleic Acids Delivery (GONAD)” and shown that this method bypasses all of the three complex-steps involved in mouse transgenesis.

## Discussion

One-step production of KO animals by CRISPR/Cas9-based genome editing through microinjection of NAs into cytoplasm and/or nuclei of zygotes is now being widely used as an alternative to traditional embryonic stem cell targeting-based KO mouse production^3^,^4^,^7^,^8^. However, it requires *ex vivo* handling of zygotes, that necessitates three important and complex procedures: i) isolation of zygotes from the female reproductive tracts, ii) microinjection of CRISPR/Cas9 NAs into the zygotes, and iii) transfer of the injected zygotes into pseudopregnant mice for their further development. These steps require sophisticated and expensive equipment and well-trained and highly-skilled personnel to perform the procedures. Such complex steps prevent researchers to readily adopt the CRISPR/Cas9 system and efficiently use it to create gene-modified organisms of their choice. Therefore, there is an urgent need for development of simpler methods to create genetically modified animal models. The method we developed here precisely accomplishes this important goal and bypasses the three critical and complex steps involved in generating gene-modified organisms. In contrast to the specialized set-up needed in traditional microinjection methods, the GONAD system requires an electroporator to deliver DNA/RNA to the embryos.

Recently, electroporation-mediated *in vitro* transfection of isolated pre-implantation embryos with CRISPR/Cas9 RNAs has been reported in rats^5^. Although they could by-pass the microinjection step, it other two steps (isolation of embryos and embryo transfer) of animal transgenesis could not be excluded from their method. In contrast, the GONAD method described here by-passes all the three major steps of animal transgenesis. Furthermore, GONAD approach is a simpler and cheaper method because it uses only two sets of animals (stud males and super-ovulated females), in contrast to all the available methods require four sets (embryo donors, stud males, embryo recipients and vasectomized males). Therefore, GONAD is more aligned with animal welfare concepts outlined in the 3R’s (Replacement, Refinement and Reduction) and should be attractive to researchers. Further, it is also more cost-effective. Another advantage of GONAD, when compared to the method described by Kaneko *et al.* 2014^5^, is that GONAD uses a low volume (2 μL) and concentration (up to 1.1 μg/μL of Cas9 mRNA and 0.6 μg/μL of sgRNA) of RNA solution whereas their method used a higher RNA concentration (Cas9 mRNA 2.0 μg/μL, sgRNA 1.0 μg/μL). In general, the electroporation-based methods require significantly higher RNA concentrations compared to those required for microinjection-based methods.

One of the potential constraints of GONAD is that the electroporation process may cause physical damage to the embryos through the electric pulses generated by the electroporator. Any such damage would reduce the overall viability of gene-modified mice. For example, we observed that about half of the recovered embryos were morphologically abnormal and failed to develop further (Table 1, Supplementary Table S2, some embryos in Supplementary Fig. S5a) while most of the embryos collected from the control mice that did not undergo electroporation developed normally to blastocysts (Supplementary Table S2) except for one C57BL/6N control that had many abnormal embryos (C-#14 in Supplementary Table S3). Even though it is premature to draw any conclusions about the genetic background being one of the factors affecting the survival rate of embryos subjected to GONAD procedure, it should be noted that C57BL/6 derived zygotes and embryos are generally more fragile than other strains as noted in the literature^9^,^10^. Further optimization of electroporation conditions, by varying electric strength and pulse durations, may help reduce the electrophoretic damage to the embryos. We anticipate that such optimization experiments would need monitoring of the pregnancy to reduce the burden on the mother if the number of implanted embryos is more than the normal range. Alternatively, the superovulation step can be omitted to prevent such situations.

The T7E1 assay identifies a sample as positive only if it contains mixture of two or more different *alleles* at the target site; either one wild-type allele along with one or more *indel* mutations containing alleles or at least two different kinds of *indel* mutations if the wild-type allele is absent. The observation that the fetuses 1 and 4 in Fig. 3 were negative by T7E1 assay, even though there were *indel* mutations in them (as revealed by sequencing), indicates that the mutation in all genomes of these two samples were of same kind. The same type of *indel* mutation in each fetus may have occurred due to one of the two possibilities. 1) The first possibility is that one of the two blastomeres of 2-cell stage zygotes might have got damaged or killed during electroporation, and the remaining undamaged blastomere (with the micro-deletion mutation) contributed to all the cells in the developed embryo. 2) The other possibility is that, same type of *indel* mutation may have occurred on different sets of genomes (coming from the two cells in the embryo) during the non-homologous end-joining repair process. Even though it is hard to demonstrate experimentally, we presume that the chances of the first possibility occurring is higher than the second one because the odds of occurrence of similar type of *indel* mutations, in both the blastomeres of 2-cell stage in two different fetuses, seems very less.

Based on this result, we conclude that the T7E1 assay can miss identifying *indel* mutations if mutations on all of the alleles in a sample are same kind (or if they are non-mosaic). In other words, if a sample is T7E1 negative, it does not mean that the sample may not contain *indel* mutations. Therefore, if T7E1 assay does not reveal any positive samples in a CRISPR/Cas9 mutagenesis experiment, it is prudent to sequence the target sites of samples to rule out the presence of *indel* mutations. An alternative strategy to identify hidden *indels* could be by mixing the PCR product of a test sample with that of a wild-type PCR product (amplified from the parental strain, for example) and subjecting the mixture to T7E1 assay.

In our previous study, we noticed mosaic expressions in most of the embryos when we electroporated plasmid DNA encoding eGFP^2^. In contrast, the electroporation of eGFP mRNA, in this study, resulted in a more uniform expression of eGFP in embryos (Fig. 2c). This may be because cytoplasmic delivery is sufficient for mRNA to be expressed, whereas delivery to the nuclei is essential for the plasmid DNA expression. Another possibility of non-mosaicism is that if one blastomere of 2-cell stage zygotes gets damaged, such embryos do not develop further and only those embryos that develop further will be from undamaged blastomere in 2-cell stage zygotes as discussed above.

In conclusion, we developed a method called GONAD that can bypass the three major and critical steps of mouse transgenesis. Specifically, this method allows direct delivery of NA reagents to the pre-implantation embryos without ever taking the embryos out from the female reproductive system. Using the GONAD system, we demonstrate that i) desired proteins can be expressed by direct delivery of mRNA and ii) genome editing can be achieved by delivering CRISPR/Cas9 tools. The GONAD system may be used for other purposes such as removal of floxed allele or FRTed allele in the KO/Tg animals by delivering Cre or FLP mRNA and knocking-in of desired sequences in the target locus. This system can also be potentially applied to generate genetically engineered models in other species including rats, pigs, sheep, goat and calves. This feature is of high significance currently because of the CRISPR/Cas9 system, which has enabled genome editing possible in many species. Over the last three decades, the transgenic technology has evolved around mice and the models were generated at core facility settings in majority of academic institutes across the world. With the introduction of CRISPR/Cas9 system, many core facilities are attempting to extend the services to other species, but such an expansion requires training to handle embryos from multiple species besides mice. The GONAD system precisely helps in this situation as it bypasses the steps of embryo isolation, handling, microinjection and embryo transfer to pseudo-pregnant animals.

## Methods

### RNA preparation

sgGFP.NT2^11^ was used as the sgRNA target sequence against eGFP. sgRNAs targets against *Hprt* and *eEF2* genes were designed using CRISPR design^4^,^12^ and/or CHOPCHOP^13^. The sgRNA listed as highly potential target in one or both the programs were chosen. The templates for sgRNA synthesis were PCR-amplified using KOD-Plus-Neo (TOYOBO, Tokyo, Japan) with primer sets (M940/M939 for eGFP, PP105/M939 for eEF2_Cr1, and PP106/M939 for eEF2_Cr2, Supplementary Table S4) using pUC57-sgRNA vector as template (Addgene plasmid number: #51132)^14^ and with primer set (M938/M939 for Hprt_Cr1) using pUC57-sgRNA-Hprt_Cr1 (target: GTCTACATATGTGCGCTACC) as template. sgRNAs were synthesized using 400 to 500 ng of gel and phenol-purified PCR products as templates and MEGAshortscript™ T7 Kit (Ambion, Austin, USA). The mRNAs were *in vitro* transcribed using *Xba* I-linearized and purified pcDNA3.1EGFP-poly(A83) (for eGFP^15^) and pBGK (for Cas9^4^) plasmids as templates and the mMESSAGE mMACHINE T7 Ultra Kit (Ambion). All RNAs were purified using MEGAclear Kit (Ambion).

### Mice

All mice were maintained at the Center of Genetic Engineering for Human Diseases (CGEHD) animal facility at Tokai University School of Medicine. ICR (10 to 18 weeks) and C57BL/6N (8 to 12 weeks) mice were obtained from CLEA Japan, Inc. (Tokyo, Japan). eGFP Tg mouse line, that contains a single copy eGFP transgene at the *Rosa26* locus, was previously generated in our facility^6^. All the animal experiments were performed in accordance with institutional guidelines and were approved by The Institutional Animal Care and Use Committee at Tokai University (Permit Number: #143037).

### Electroporation into embryos within the dissected oviducts

The super-ovulated C57BL/6N female mice were mated to adult male C57BL/6N mice or eGFP Tg^9^ (homozygous for the transgene) males. Presence of copulation plugs was confirmed by visual inspection next morning and the females having plugs were designated as day 0.5 of gestation and used for the electroporation experiments.

The solution contained *in vitro*-synthesized RNAs (concentrations listed in Supplementary Table S3) and 0.05% of trypan blue (Nacalai tesque Inc., Kyoto, Japan) used as a marker for successful instillation, prepared in Nuclease-Free Water (Ambion). Instillation of 2 μL of this solution into the lumen of the oviducts, dissected from the plugged female mice (day 1.5), was performed through the oviductal wall near the infundibulum using a mouth piece-controlled glass micropipette (Step 3 of Fig. 1). After instillation, the RNA-containing oviducts were transferred to 80 μL of Hepes-buffered saline (HBS; Applichem, Darmstadt, Germany) in 1-mm Gap cuvettes (BTX Electroporation Cuvettes Plus; BTX Genetronics Inc., San Diego, CA, USA). The electroporation was performed using a square-wave pulse generator (T820; BTX Genetronics Inc.). The electroporation parameters were as follows; eight square-wave pulses with a pulse duration of 5 ms and an electric field intensity of 50 V. Our understanding is that the T820 model BTX electroporator is no longer manufactured and we suggest an equivalent model that will deliver the same pulse. After the electroporation, the embryos were recovered by flushing oviducts with M2 Medium (Sigma-Aldrich Co., St Louis, MO, USA), and cultured in KSOM medium (ARK Resource, Kumamoto, Japan) until analyzed. The embryos isolated from the dissected, but non-electroporated oviducts were used as controls.

### Electroporation into oviducts *in situ*

Preparation of reagents and mice (C57BL/6N or ICR) for electroporation was as described in the *in vitro* electroporation. Females on Day 1.5 (corresponding to 2-cell stage) were anesthetized using 20% isoflurane in propylene glycol (as shown in Step 1 of Fig. 1). The anesthetized mice were placed onto a Micro-warm-plate (KM-1; Kitazato Co., Tokyo, Japan) at 37 °C and surgical procedures were performed under observation using a dissecting microscope (SZST; Olympus, Tokyo, Japan), as described below.

The ovary/oviduct/uterus were exposed after making an incision at the dorsal skin (Step 2 of Fig. 1). The oviducts were located and positioned suitably for further steps. Approximately 2 μL of RNA solution was slowly injected into the oviductal lumen near the infundibulum using a micropipette that were prepared using an electric puller (P-97/IVF; Sutter Instrument Co., Novato, CA, USA) with a mouthpiece attached (Step 3 of Fig. 1). The micropipette was rapidly retracted after completing the injection procedure. Immediately after the injection of electroporation solution, the oviductal regions were covered with a small piece of wet paper (Kim wipe; Jujo-Kimberly Co. Ltd., Tokyo, Japan) soaked in phosphate-buffered saline (PBS), and were grasped in tweezer-type electrodes, with disc electrodes (diameter, 5 mm) at their tips (#CT-234; NEPA GENE Co. Ltd., Ichikawa, Chiba, Japan) (Step 3 of Fig. 1). The electroporation conditions were as described in the *in vitro* electroporation. The gaps between the electrodes used to hold the oviduct were approximately 2 mm. After the electroporation, the oviducts were returned to their original position (Step 4 of Fig. 1) and the incisions were sutured. The animals were monitored for anesthesia recovery and were housed until the embryos were harvested for further analysis.

### Observation of eGFP fluorescence

The dissected oviducts and embryos were observed using a fluorescence microscope (BIOREVO BZ-9000; Keyence, Osaka, Japan) for detecting the eGFP fluorescence. Oviducts exhibiting fluorescence are defined as + (Table 1 and Supplementary Table S2). The intensity of fluorescence was assessed by comparing with the control embryos/fetuses of the same developmental stage. Fluorescence in fetuses was observed using a fluorescence stereomicroscope (M165FC; Leica Microsystems, Germany).

### Analysis of CRISPR/Cas9-induced mutations

Genomic DNAs were isolated from the single blastocysts or the tail-piece of fetus using All-In-One Mouse Tail Lysis Buffer (ABP-PP-MT01500; KURABO, Osaka, Japan) through incubation at 60 °C for 3 h and subsequent inactivation at 95 °C for 10 min. The 1^st^ PCR for amplification of a target locus was performed in a 10 μL of 1X PCR buffer containing genomic DNA (1 μL), primers (Supplementary Table S4) and 0.25 U/μL of TaKaRa LA-Taq (TaKaRa Biomedicals, Otsu, Japan) using denaturation (96 °C for 1 min), 24 cycles of 96 °C for 30 sec, 66 °C for 1.5 min, and extension (68 °C for 7 min). The nested PCR was then performed in a 10 μL of PCR buffer containing 0.5 μL of 1^st^ PCR products (100X dilution was used for *eEF2*), primers (listed in Supplementary Table S4) and 0.25 U/μL of TaKaRa r-Taq (TaKaRa Biomedicals) using denaturation (95 °C for 5 min), 35 cycles of 95 °C for 45 sec, 58 °C for 30 sec and 72 °C for 1 min, and extension (72 °C for 5 min).

Surveyor assays were performed using 5 μL of a solution containing nested PCR products (derived from experimental samples) with a SURVEYOR Mutation Detection Kit (Transgenomic, Omaha, NE, USA) following the protocol specified by the manufacturer. T7E1 assays were performed using 5 μL of the above solution mixed with 1 μL of 10X NEB Buffer 2 (New England Biolabs Japan Inc., Tokyo, Japan), 0.2 μL of T7E1 (2 unit; NEB) and 3.8 μL of distilled water and incubated the mixture at 37 °C for 30 min. The reaction products were then analyzed in 2% agarose gels and stained with SYBR Gold (Life Technologies Japan, Tokyo, Japan).

Direct sequencing was performed using the purified PCR products and the primers listed in Supplementary Table S4. For accurate determination of *indel* mutations in fetuses 2 and 3 in Fig. 3, PCR-amplified fragments were cloned into a TA Cloning vector contained in the TOPO® TA Cloning Kit (Life Technologies), and the insert sequences of 18 (for fetus 2) to 10 (for fetus 3) colonies were determined.

## Additional Information

**How to cite this article**: Takahashi, G. *et al.* GONAD: *G*enome-editing via *O*viductal *N*ucleic *A*cids *D*elivery system: a novel microinjection independent genome engineering method in mice. *Sci. Rep.* **5**, 11406; doi: 10.1038/srep11406 (2015).

## Supplementary Material

Supplementary Information

## Figures and Tables

**Table 1:** Delivery of CRISPR/Cas9 components to pre-implantation embryos in oviducts *in situ *using the GONAD^1^ system.

Mice	Concentration of RNAs	Day 2.5 : 8-cell to 16-cell stages				Day 3.5 : morula to blastocyst stages			No. offluorescentembryosshowing*indel*mutation^4^
		Fluorescence of oviducts^2^	No. of embryos recovered (No. of embryos showing fluorescence)			No. of embryos developed (No. of embryos showing fluorescence)			
			Total	Normal	Abnormal^3^	Total	Normal	Abnormal^3^	
D-#01	Cas9 mRNA (1096 ng/μl) Hprt_Cr1_sgRNA (530 ng/μl) eGFP mRNA (632 ng/μl)	+	9 (3)	4 (0)	5 (3)	9 (3)	4 (0)	5 (3)	0
D-#02		+	17 (0)	11 (0)	6 (0)	17 (0)	11 (0)	6 (0)	0
D-#03	Cas9 mRNA (1096 ng/μl) Hprt_Cr1_sgRNA (469 ng/μl) eGFP mRNA (632 ng/μl)	+	20 (1)	15 (1)	5 (0)	20 (1)	14 (1)	6 (0)	1
D-#04		+	10 (3)	6 (2)	4 (1)	10 (2)	3 (0)	7 (2)	0
D-#05		+	25 (8)	7 (3)	18 (5)	25 (8)	7 (3)	18 (5)	3
D-#06		+	18 (4)	13 (2)	5 (2)	18 (4)	10 (2)	8 (2)	1
Total			99 (19)	56 (8)	43 (11)	99 (18)	49 (6)	50 (12)	5

^1^The electroporation procedure was performed into intact oviducts of C57BL/6N mice as described in the Methods. One day after electroporation, embryos were flushed from the treated oviducts and then cultured to morula or blastocyst stages.

^2^At least one oviduct of the two oviducts per mouse exhibiting fluorescence was defined as +.

^3^Abnomal embryos were defined as those showing fragmentation, developmental arrest or degeneration.

^4^*Indel* mutation analysis was performed using T7E1 assay on genomic DNA from separate embryos. Sequencing was performed to confirm the mutations in some cases.
