# Supplementary material for: GONAD: Genome-editing via Oviductal Nucleic Acids Delivery system: a novel microinjection independent genome engineering method in mice
Source: Sci Rep. 2015 Jun 22;5:11406. doi: 10.1038/srep11406 (PMC4476150; doi:10.1038/srep11406)
Supplement: Supplementary Information [file srep11406-s1.pdf]

Supplementary Information

Genome-editing via Oviductal Nucleic Acids Delivery (GONAD) system: a novel microinjection-independent genome engineering method in mice

Gou Takahashi, Channabasavaiah B Gurumurthy, Kenta Wada, Hiromi Miura, Masahiro Sato, Masato Ohtsuka

Supplementary Tables

Supplementary Table S1

Supplementary Table S1 | Delivery of eGFP mRNA to pre-implantation embryos in oviducts *in vitro*[1]

Day 1.5 : 2-cell stages			Day 2.5 : 8-cell to16-cell stages	Day 3.5 : morula to blastocyst stages
No. of embryos recoverd			No. of embryos normally developed (No. of embryos showing fluorescence)	No. of embryos normally developed (No. of embryos showing fluorescence)
Total	Normal	Abnormal[2]		
27	16	11	16 (5)	16 (4)

[1]eGFP mRNA (500 ng/μL) was electroporated into a dissected oviduct isolated from C57BL/6N mice (A-#01) as described in Methods. 2-cell embryos were flushed from the treated oviducts and cultured for two days up to morula or blastocyst stages.

[2]Abnomal embryos were defined as those showing fragmentation, developmental arrest or degeneration.

Supplementary Table S2

Supplementary Table S2 | Delivery of eGFP mRNA to pre-implantation embryos in oviducts *in situ*[1]

Group	Mice	Fluorescence of oviducts[2]	Day 2.5 : 8-cell to 16-cell stages			Day 3.5 : morula to blastocyst stages		
			No. of embryos recoverd (No. of embryos showing fluorescence)			No. of embryos developed (No. of embryos showing fluorescence)		
			Total	Normal	Abnormal[3]	Total	Normal	Abnormal[3]
Experimental group	B-#01	+	11 (1)	6 (0)	5 (1)	11 (1)	5 (0)	6 (1)
	B-#02	+	8 (6)	3 (3)	5 (3)	8 (4)	2 (2)	6 (2)
	B-#03	+	9 (1)	2 (1)	7 (0)	9 (1)	2 (1)	7 (0)
	B-#04	+	4 (0)	0 (0)	4 (0)	4 (0)	0 (0)	4 (0)
	B-#05	+	13 (5)	10 (4)	3 (1)	13 (4)	10 (3)	3 (1)
	B-#06	+	7 (0)	4 (0)	3 (0)	7 (0)	4 (0)	3 (0)
	Total		52 (13)	25 (8)	27 (5)	52 (10)	23 (6)	29 (4)
Control group	B-#07	-	15	13	2	15	13	2
	B-#08	-	17	17	0	17	17	0
	B-#09	-	14	14	0	14	14	0
	Total		46	44	2	46	44	2

[1]eGFP mRNA (500 ng/µL) was electroporated into an intact oviduct of ICR mice as described in Methods. One day after electroporation, embryos were flushed from treated oviducts and then cultured up to morula to blastocyst stages. No electroporation was performed for the control group.

[2]At least one oviduct of the two oviducts per mouse exhibiting fluorescence was defined as +.

[3]Abnormal embryos were defined those showing fragmentation, developmental arrest or degeneration.

Supplementary Table S3

Supplementary Table S3 | Delivery of CRISPR/Cas9 RNAs to pre-implantation embryos in oviducts *in vitro*[1]

Group	Mice	RNAs injected (concentration)	Day 1.5 : 2-cell stage			Day 2.5 : 8-cell to 16-cell stages	Day 3.5 : morula to blastocyst stages	No. of embryos showing *indel* mutation[3]
			No. of embryos recoverd			No. of embryos normally developed (No. of embryos showing fluorescence)	No. of embryos normally developed (No. of embryos showing fluorescence)	
			Total	Normal	Abnormal[2]			
Experimental group	C-#01	eGFP mRNA (150 ng/µl) Cas9 mRNA (500 ng/µl) Hprt_Cr1_sgRNA (50 ng/µl)	13	12	1	12 (6)	12 (6)	1
	C-#02		24	13	11	10 (1)	10 (1)	0
	C-#03		12	12	0	12 (2)	11 (2)	0
	C-#04		26	22	4	22 (5)	22 (5)	0
	C-#05		15	10	5	9 (0)	9 (0)	0
	C-#06	eGFP mRNA (200 ng/µl) Cas9 mRNA (400 ng/µl) Hprt_Cr1_sgRNA (100 ng/µl)	21	16	5	16 (1)	16 (1)	0
	C-#07		12	5	7	5 (2)	5 (2)	1
	C-#08	Cas9_mRNA (923 ng/µl) eEF2_Cr1_sgRNA (243 ng/µl)	6	6	0	6 (NA)	6 (NA)	0
	C-#09		10	10	0	10 (NA)	10 (NA)	2
	C-#10	Cas9_mRNA (500 ng/µl) eEF2_Cr2_sgRNA (409 ng/µl)	24	24	0	24 (NA)	24 (NA)	0
	C-#11		10	10	0	9 (NA)	9 (NA)	0
	C-#12	Cas9 mRNA (444 ng/µl) eGFP_sgRNA (133 ng/µl)	13	8	5	8 (NA)	5 (NA)	0
	C-#13		17	10	7	8 (NA)	7 (NA)	1
Control group	C-#14	-	16	5	11	5	5	N.D.
	C-#15		8	7	1	7	7	N.D.

[1]RNAs were electroporated into a dissected oviduct isolated from C57BL/6N mice as described in Methods. 2-cell embryos were flushed from treated oviducts and then cultured for two days up to morula to blastocyst stages. No electroporation was performed for the control group.

[2]Abnomal embryos were defined those showing fragmentation, developmental arrest or degeneration.

[3]*Indel* mutation analysis was performed using Surveyor- or T7E1-assay from genomic DNA of separate embryos. Sequencing was performed to confirm the mutations in some cases. N.D.= not done.

Supplementary Table S4

Supplementary Table S4 | Primer sets for sgRNA synthesis, Surveyor- and T7E1-assay

	Target		Primer name	Primer sequence (5' to 3')	Product size
Primer sets for sgRNA synthesis	eGFP	F	M940	TAATACGACTCACTATAGGGACC AGGATGGGCACCACCCGTTTTA GAGCTAGAAATAGCAAG	122-bp
		R	M939	AAAAAAAGCACCGACTCGG	
	Hprt_Cr1	F	M938	TAATACGACTCACTATAGG	122-bp
		R	M939	AAAAAAAGCACCGACTCGG	
	eEF2_Cr1	F	PP105	TAATACGACTCACTATAGGGCTA CCGGAAAGGGCCCGGAGTTTTA GAGCTAGAAATAGCAAG	122-bp
		R	M939	AAAAAAAGCACCGACTCGG	
	eEF2_Cr2	F	PP106	TAATACGACTCACTATAGGGGGA GCGACTTCCCAAGGCTGTTTTA GAGCTAGAAATAGCAAG	122-bp
		R	M939	AAAAAAAGCACCGACTCGG	
Primer sets for Surveyor- and T7E1-assay	eGFP_1st PCR	1F	M212	CTCCTGGGCAACGTGCTGGT	376-bp
		1R	M495	AAGAAGATGGTGCGCTCCTG	
	eGFP_2nd PCR	2F	M389	TCGCCACCATGGTGAGCAAGG GCGAG	162-bp
		2R	M026*	GGTGGTGCAGATGAACTTCAG	
	Hprt_Cr1_1st PCR	1F	M925	TGATGCAGGACGATTTCAAG	609-bp
		1R	M926	GGATGGTTGTGAGCCATGAT	
	Hprt_Cr1_2nd PCR	2F	M949*	GCCAGCCAGATCCTATCTAAA	445-bp
		2R	M950	TGACAAGAAGATGGGAATGG	
	eEF2_Cr1_1st PCR	1F	PP113	ATATCTGCGCGTCCCTGA	525-bp
		1R	PP114	TGAGGCTCAAGTCACATAACC	
	eEF2_Cr1_2nd PCR	2F	PP115*	TTGTGCTTGGTGATGTGG	301-bp
		2R	PP116	CACACACCTCTTCCCCAAA	
	eEF2_Cr2_1st PCR	1F	PP117	TGGGGAGACCGGTGAGTA	527-bp
		1R	PP118	GAGGCCCCTCGTATAGCA	
	eEF2_Cr2_2nd PCR	2F	PP119	GCCAATGGCAAGTTCAGTA	301-bp
		2R	PP120	GCCTTGAGCAATGGCTTG	

*Primers used for direct sequencing.

Supplementary Figures

Supplementary Figure S1

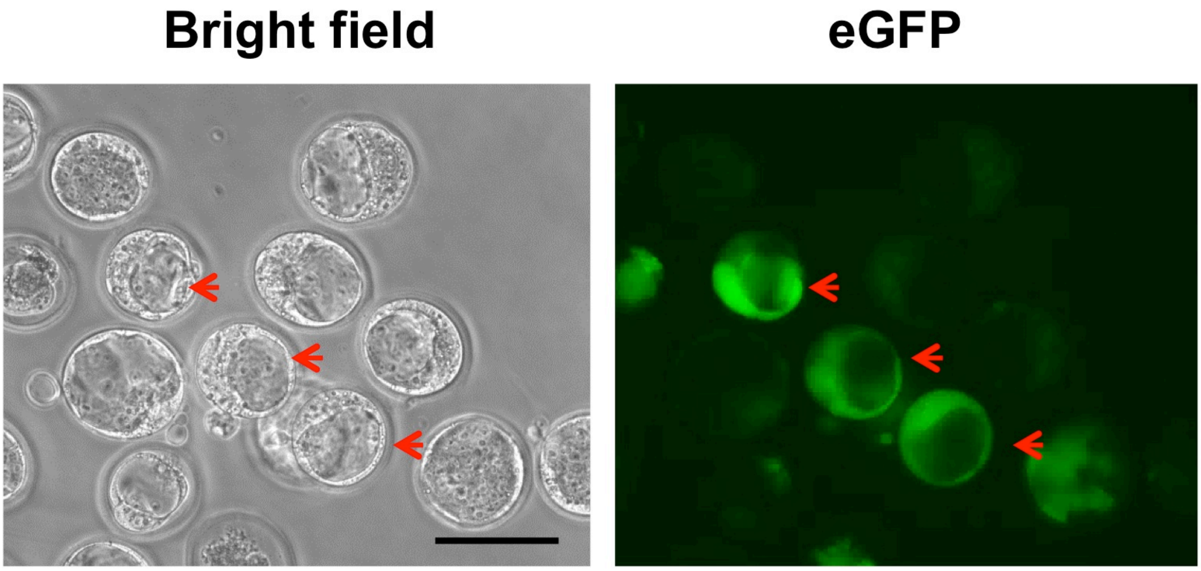

Supplementary Figure S1 | eGFP expression in 8- to 16-cell stage embryos recovered from the dissected oviducts that were *in vitro* electroporated with eGFP mRNA. The embryos were derived from A-#01 mouse (C57BL/6N strain) shown in Supplementary Table S1. The embryos exhibiting fluorescence throughout are shown with red arrows. Scale bar = 100 μm.

Supplementary Figure S2

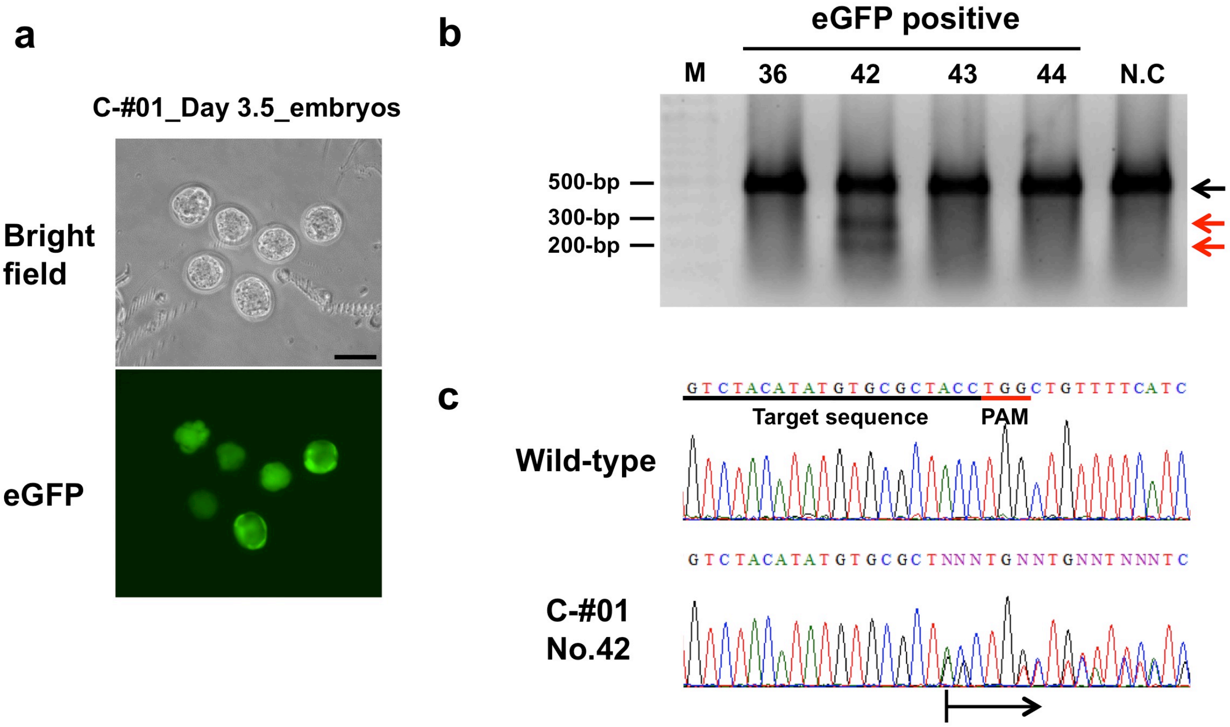

Supplementary Figure S2 | CRISPR/Cas9-mediated disruption of *Hprt* locus achieved through *in vitro* electroporation of oviducts. (a) eGFP fluorescence in the blastocysts, one day after culturing of embryos recovered from an *in vitro* electroporated mouse with eGFP mRNA (used as an indicator of electroporation), Cas9 mRNA and Hprt_Cr1 sgRNA (C-#01 mouse: Supplementary Table S3). Scale bar = 100 µm. (b) Agarose gel electrophoresis of a few Surveyor-treated PCR products derived from eGFP fluorescence-positive embryos (C-#01_No.36, 42, 43 and 44). The red arrows indicate the cleavage products generated in Surveyor assay and the wild-type sized band is indicated by a black arrow M: 100-bp DNA ladder markers. N.C: a Negative Control embryo derived from a non-instilled and non-electroporated mouse. (c) Direct sequencing of the PCR products from wild-type mouse or a mutated blastocyst (C-#01_No.42, shown in (b)). The black arrow below the electropherogram shows overlapping peaks indicative of *indel* mutations (see text for details).

Supplementary Figure S3

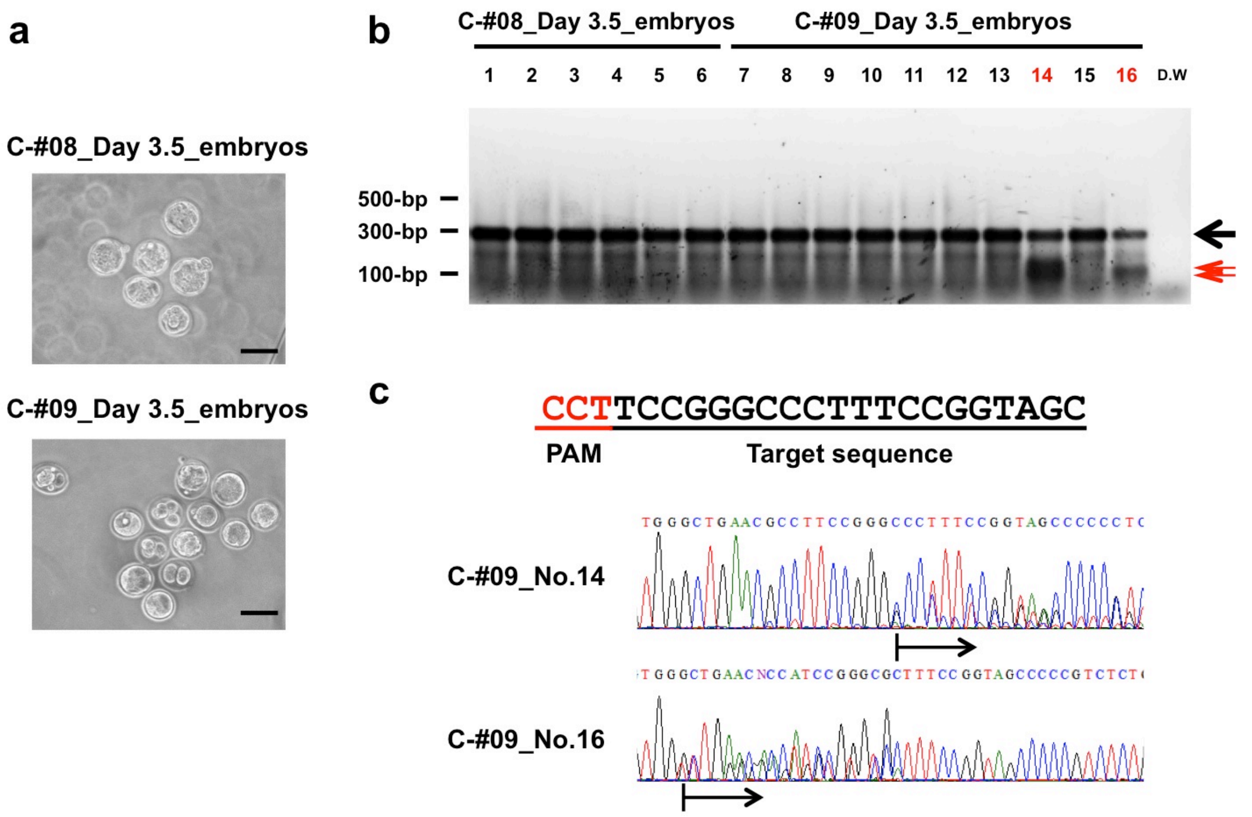

Supplementary Figure S3 | CRISPR/Cas9-mediated disruption of *eEF2* locus achieved through *in vitro* electroporation of oviducts. (a) Bright field micrograph of embryos collected and cultured for two-days after *in vitro* electroporation of CRISPR/Cas9 RNAs against the *eEF2* gene (eGFP mRNA, as an indicator of electroporation, was not included in this experiment). The embryos from two different mice (C57BL/6N strain) are shown: the upper panel embryos were recovered from the C-#08 mouse (Supplementary Table S3) and the bottom panel embryos were recovered from the C-#09 mouse. Scale bars = 100 μm. (b) Agarose gel electrophoresis of T7E1-treated PCR products derived from the embryos shown in (a). The red arrows indicate the cleavage products generated in T7E1 assay and the wild-type sized band is indicated by a black arrow. D.W: distilled water used as negative control. M: 100-bp DNA Ladder. (c) Direct sequencing of the PCR products of the target region amplified from samples C-#09_No.14 and _No.16. The black arrow below the electropherogram shows overlapping peaks indicative of *indel* mutations (see text for details).

Supplementary Figure S4

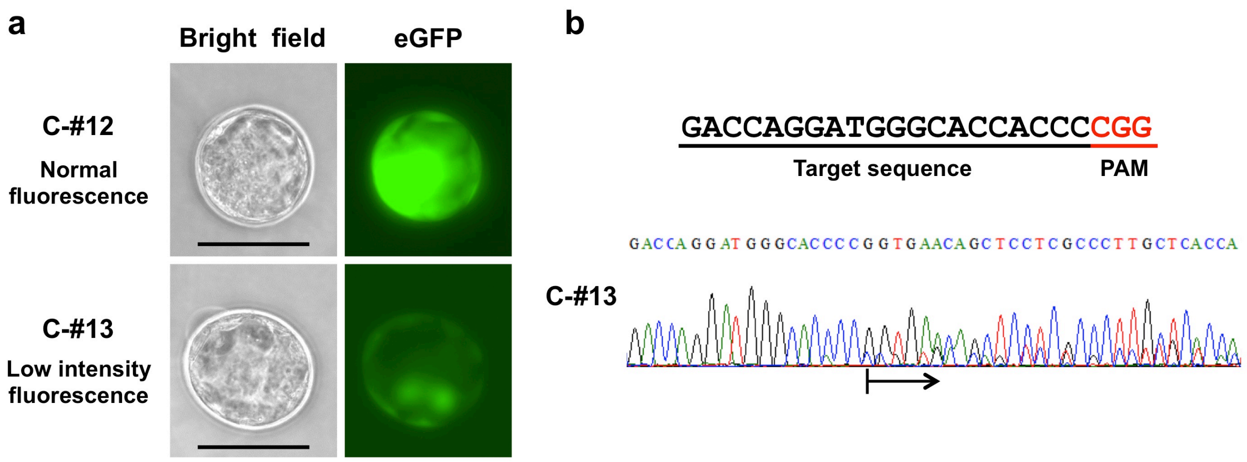

Supplementary Figure S4 | CRISPR/Cas9-mediated disruption of eGFP transgene achieved through *in vitro* electroporation of oviducts. (a) eGFP fluorescence in the blastocysts, two-days after culturing of embryos recovered from an *in vitro* electroporated mouse (C57BL/6N strain mated with hemizygote eGFP Tg males) with Cas9 mRNA, and eGFP sgRNA. One embryos each from two different mice are shown: the upper panel embryo showing normal eGFP fluorescence recovered from the C-#12 mouse (Supplementary Table S3) and the bottom panel embryo showing greatly reduced fluorescence was recovered from the C-#13 mouse. Scale bars: 100 μm. (b) Direct sequencing of the PCR product amplified from the target region from bottom panel's embryo in (a). The black arrow below the electropherogram shows overlapping peaks indicative of *indel* mutations (see text for details).

Supplementary Figure S5

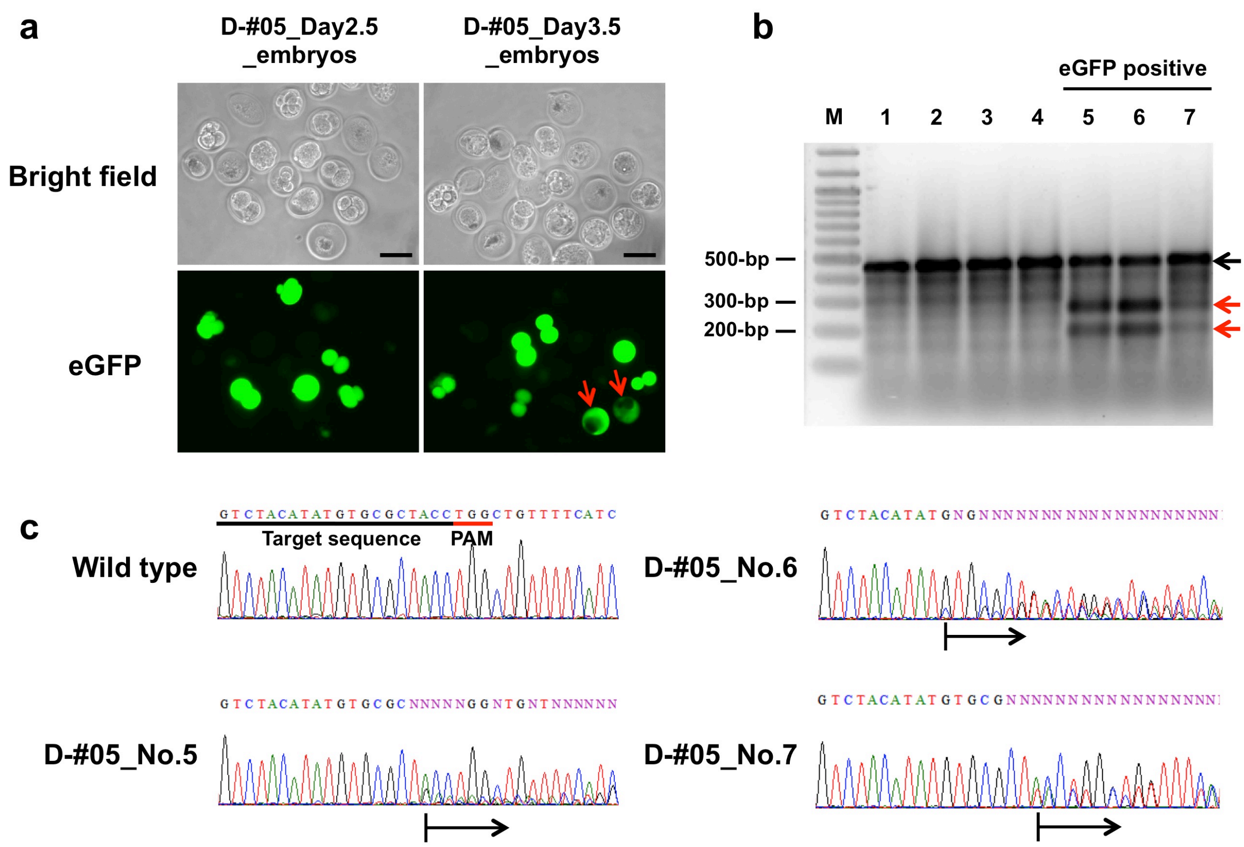

Supplementary Figure S5 | CRISPR/Cas9 mediated disruption of *Hprt* locus using the GONAD system. (a) eGFP fluorescence at the 8-cell to 16-cell embryos (left side panels) and blastocyst embryos (right side panels) that were collected from GONAD procedure performed using eGFP mRNA (used as an indicator of electroporation), Cas9 mRNA and Hprt_Cr1 sgRNA (Table 1). Scale bars = 100 µm. (b) Agarose gel electrophoresis of T7E1-treated PCR products derived from 7 selected blastocysts. The red arrows indicate the cleavage products generated in T7E1 assay and the wild-type sized band is indicated by a black arrow. M: 100-bp DNA ladder marker. (c) Direct sequencing of PCR products amplified from the target region from wild-type, D-#05_No.5, _No.6, and _No.7 samples. The black arrow below the electropherogram shows overlapping peaks indicative of *indel* mutations (see text for details).

Supplementary Figure S6

Founder no.	Target sequence / PAM	
	TTACGTCGCCGTCCAGCTCGACCAGGATGGGCACCACCCCGGTGAACAGCTCCTCGCCCTT	WT
1 (Fig.3)	TTACGTCGCCGTCCAGCTCGACCAGGATGGGCACC----CGGTGAACAGCTCCTCGCCCTT	Δ4
2 (Fig.3)	TTACGTCGCCGTCCAGCTCGACCAGGATGGGC------CCGGTGAACAGCTCCTCGCCCTT	Δ6
3 (Fig.3)	TTACGTCGCCGTCCAGCTCGACCAGGATGGGCACC---CCGGTGAACAGCTCCTCGCCCTT	Δ3
4 (Fig.3)	TTACGTCGCCGTCCAGCTCGACCAGGATGGGCACC---CCGGTGAACAGCTCCTCGCCCTT	Δ3
3 (Fig.4)	TTACGTCGCCGTCCAGCTCGACCAGGATGGGCACC---CCGGTGAACAGCTCCTCGCCCTT	Δ3
7 (Fig.4)	TTACGTCGCCGTCCAGCTCGACCAGGATGGGCACCAACCCCGGTGAACAGCTCCTCGCCCTT	+1
	TTACGTCGCCGTCCAGCTCGACCAGGATGGGCACC----CGGTGAACAGCTCCTCGCCCTT	Δ4

Figure S6 | Mutated eGFP alleles possessed in founder fetuses. The changes in the nucleotide sequence are shown in red and the type of changes (insertions: + or deletions: Δ) are indicated on the right-side of the sequences. All fetuses had only one type of mutation except the fetus 7 of Figure 4 which was a mosaic with two mutant alleles (shown in a box).
